# Neutrophil to lymphocyte ratio may predict efficacy of anti-PD-1 inhibitors in advanced EGFR-mutant non-small cell lung cancer: retrospective cohort study

**DOI:** 10.1038/s41598-024-54557-0

**Published:** 2024-02-20

**Authors:** Jianxin Chen, Qinhong Zheng, Shijian Zhu, Dan Qiu, Junhui Wang

**Affiliations:** 1 Department of Medical Oncology, The Quzhou Affiliated Hospital of Wenzhou Medical University, Quzhou People’s Hospital, Quzhou, 324000 Zhejiang China; 2 Department of Radiation Oncology, The Quzhou Affiliated Hospital of Wenzhou Medical University, Quzhou People’s Hospital, Quzhou, 324000 Zhejiang China

**Keywords:** Non-small cell lung cancer, Epidermal growth factor receptor, Anti-PD-1 inhibitor, Neutrophil to lymphocyte ratio, Efficacy, Immunotherapy, Cancer therapy, Lung cancer

## Abstract

This study aimed to investigate the associations between the clinical characteristics and effectiveness of anti-PD-1 inhibitors in patients with EGFR-sensitive mutations, aiming to identify the potential subgroup of patients who might benefit from anti-PD-1 inhibitor treatment. Patients with advanced non-small cell lung cancer (NSCLC) harboring epidermal growth factor receptor (EGFR)-sensitive mutations who received subsequent anti-PD-1 inhibitors in combination with chemotherapy/antiangiogenic agents or alone after progression to tyrosine kinase inhibitors (TKIs) were screened. Clinical characteristics, including hematological parameters, were investigated for potential correlations with clinical outcomes. Subgroup and multivariate analyses were used for further confirmation of the relationship. Kaplan–Meier curves and Cox survival regression models using the log-rank test were used for progression-free survival (PFS) and overall survival (OS) assessments between the groups. Multiple regression analysis was performed using the standard regression coefficient values. The Wilcoxon test was used for the analysis of the variation in NLR*. P* ≤ 0.05 was considered to indicate statistical significance. This study was a retrospective study. Twenty-two patients met the inclusion criteria and were included in the study. The median PFS was 3.05 months (95% CI, 2.9–10.2 months). The median OS was 7.30 months (95% CI, 5.2–18.1 months). PFS in low neutrophil to lymphocyte ratio (NLR ≤ 4) was significantly longer than high NLR (NLR > 4, 5.7 months versus 2.0 months, HR, 0.35, 95% CI, 0.08–0.63, *P* = 0.0083). The OS in the low NLR group was also significantly better than that in the high NLR group (OS, 21.3 months versus 5.0 months, HR, 0.33; 95% CI, 0.09–0.74; *P* = 0.0163). In the multivariate analysis, NLR was the only significant factor for OS benefits (β = 3.535, 95% CI, 1.175–10.636, *P* = 0.025). Further investigation revealed that front-line TKIs exposure may contribute to the elevation or decrease of NLR, and finally lead to different efficacy outcomes by anti-PD-1 inhibitors. The findings suggest that a portion of advanced NSCLC patients with low NLR characteristics (NLR ≤ 4), even those harboring EGFR-sensitive mutations, could benefit from anti-PD-1 inhibitors as further line treatment after progression to EGFR-TKIs.

## Introduction

Immune checkpoint inhibitors (ICIs), including anti-programmed death-1 (PD-1) and programmed deathligand-1 (PD-L1) inhibitors, have been approved as treatment strategies for various cancer types, including non-small cell lung cancer, owing to their promising efficacy^1–3^. However, the efficacy of ICIs in patients with non-small cell lung cancer (NSCLC) harboring epidermal growth factor receptor (EGFR)-sensitive mutations (exon 19 deletion/exon 21 L858R mutation) is disappointing and usually results in severe adverse events^3–6^. Regimens, including cytotoxic agents with or without antiangiogenic drugs, have been recommended as standard options for patients with EGFR-sensitive mutations who have progressed on tyrosine kinase inhibitors (TKIs), which lead to limited efficacy and distinct toxicities.

Even so, there still have several literatures reporting that ICIs in combination with chemotherapy or antiangiogenic agents, demonstrating promising efficacy in recent years^7–9^. The trial IMpower150 was conducted to investigate the efficacy of atezolizumab plus bevacizumab plus carboplatin plus paclitaxel (ABCP) versus the standard regimen of bevacizumab plus carboplatin plus paclitaxel (BCP) in chemotherapy-naive patients with non-squamous NSCLC, in which a small number of EGFR-mutant patients had progressed to TKIs. The results of subgroup analysis showed significant benefits on overall survival (OS) with ABCP versus BCP in patients with sensitive EGFR mutations (median OS NE (95% CI NE-NE) with ABCP versus 17.5 months (95% CI 11.7-NE) with BCP, HR, 0.31, 95% CI 0.11–0.83), suggesting potential clinical benefits for ICIs, even in EGFR mutant patients^7^. In addition, another randomized phase II study was performed to evaluate the feasibility of combination treatment with pembrolizumab plus docetaxel in patients with pre-treated advanced NSCLC following chemotherapy regardless of EGFR mutations^8^. Subgroup analysis of EGFR-sensitive mutation patients still revealed considerable improvement in ORR (58.3% versus 23.1%, *P* = 0.14) and progression-free survival (PFS)(6.8 months, 95% CI, 6.2-not reached) versus 3.5 months, 95% CI, 2.3–6.2, *P* = 0.04)^8^. Based on these findings, we considered that a portion of EGFR-mutant patients may respond to ICIs treatment, which was also observed in our clinical practice. However, identification of such a group of patients who potentially respond to ICIs is an urgent challenge.

Hence in the current research, the associations between clinical characteristics and effectiveness of anti-PD-1 inhibitors in advanced NSCLC patients harboring EGFR sensitive mutations were investigated within a retrospective real-world approach.

## Method

### Data source

The data of patients diagnosed with advanced NSCLC harboring EGFR sensitive mutation (exon 19 deletion/exon 21 L858R mutation) in Quzhou People’s Hospital, between January 2019 and February 2022, were retrieved from the electronic medical record system. After progression to EGFR-TKIs without any sensitive acquired mutations, patients who received subsequent anti-PD-1 inhibitors, including pembrolizumab, sintilimab, tislelizumab, and camrelizumab in combination with chemotherapy/antiangiogenic agents or anti-PD-1 inhibitors alone as salvage treatment, were further screened. Data and follow-up records were updated on April 30, 2022. All the included patients had at least one measurable lesion. This study was approved by the ethical committee of the People’s Hospital of Quzhou. All investigations in the present study were performed in accordance with the Declaration of Helsinki (revised in 2013).

### Patient selection and outcomes evaluation

Patients were considered eligible to be included in the present retrospective real-world study if they met the following inclusion criteria: (1) definitive histological or cytological diagnosis of advanced NSCLC; (2) patients harboring EGFR sensitive mutations (exon 19 deletion/exon 21 L858R mutation) receiving EGFR-TKIs as a front-line treatment; (3) progressed EGFR-TKIs without any sensitive mutations detected; (4) patients receiving subsequent anti-PD-1 inhibitors including pembrolizumab, sintilimab, tislelizumab, and camrelizumab in combination with chemotherapy/antiangiogenic agents or anti-PD-1 inhibitors alone as salvage treatment. The exclusion criteria were as follows: (1) a history of autoimmune disease; and (2) a poor ECOG performance status of > 2; (3) without measurable lesions.

The clinical response to anti-PD-1 inhibitors in combination with chemotherapy/antiangiogenic agents or anti-PD-1 inhibitors alone was evaluated according to the Response Evaluation Criteria in Solid Tumors (RECIST) version 1.1. The included patients underwent imaging evaluation every 6–8 weeks during treatment. The objective response rate (ORR) was defined as the percentage of patients who achieved a complete response (CR: complete remission of all target lesions) or partial response (PR: at least a 30% reduction in the sum of the diameters of target lesions). Progressive disease (PD) referred to a 20% increase in the sum of the diameters of target lesions. A disease that could not be classified as PR or PD was evaluated as stable disease (SD). The percentage of patients with CR, PR, or SD was defined as the disease control rate (DCR). Duration of response (DoR) refers to the time from the first PR to the first PD among patients who had been evaluated as PR. PFS was calculated as the time from the initiation of anti-PD-1 inhibitor treatment to PD or death. OS referred to the time from the initiation of anti-PD-1 inhibitor treatment to PD or death. NLR was tested in two weeks before the first dose of anti-PD-1 inhibitors. High NLR referred to neutrophil to lymphocyte ratio > 4, while low NLR ≤ 4. TKI-PFS was identified as the total PFS, which included all progression-free survival time by all kinds of TKIs before exposure to anti-PD-1 inhibitors. Long-term TKI-PFS was defined as PFS > 10 months and short-term one less < 10 months, which were included in the multivariate analysis. Adverse events (AEs) were graded according to the National Cancer Institute Common Terminology Criteria for Adverse Events version 4.0 (NCI-CTCAE v4.0).

### Statistical analysis

Descriptive statistics (percentages, means, and medians) were used to describe the baseline characteristics and clinical features of the patients with advanced NSCLC. Short-term efficacy was evaluated using ORR and DCR. Kaplan–Meier curves and Cox survival regression models using the log-rank test were adopted for PFS and OS assessment between the groups with high and low NLR. K-M curves were plotted using GraphPad Prism 9.0 (GraphPad Software Inc., San Diego, CA, USA). Multiple regression analysis was performed with standard regression coefficient values using SPSS (version 22.0; SPSS Inc., Chicago, IL, USA). The variation in NLR did not pass the normal distribution test. The Wilcoxon test was used for the analysis of the variation in NLR*. P* ≤ 0.05 was considered to indicate statistical significance.

### Ethical approval

This study was approved by the ethical committee of the People’s Hospital of Quzhou. All investigations in the present study were performed in accordance with the Declaration of Helsinki (revised in 2013).

### Ethics statements

The publication of the present study details was approved by Ethical Committee of People’s Hospital of Quzhou. Written informed consent was obtained from the patients or their relatives for publication of this study and any accompanying images.

## Results

### Patient characteristics

From January 2019 to February 2022, 274 patients with advanced NSCLC harboring EGFR-sensitive mutations (exon 19 deletion/exon 21 L858R mutation) were screened using the electronic medical record system. After progression to EGFR-TKIs without subsequently detected alterations, 22 patients received anti-PD-1 inhibitors alone or in combination with chemotherapy/angiogenesis as salvage treatment. The baseline characteristics and clinical features of the patients are presented in Table 1. The median age of eligible patients was 58 years. The majority of screened patients (90.9%) were diagnosed with adenocarcinoma. Fourteen patients were with ECOG PS status of 0 (63.6%), seven (31.8%) patients with ECOG PS status of 1, and one patient (4.6%) with that of 2 at the time of diagnosis. Thirteen patients (59.1%) were current or former smokers. Fifteen (68.2%) patients had a standard BMI index from 18.5 to 23.9, with two patients (9.1%) having a BMI < 18.5, and five (22.7%) patients had a BMI > 24. In the present study, twelve patients (54.5%) had EGFR exon 19 deletions, leaving ten patients (45.5%) with the EGFR exon 21 L858R mutation. As presented, eight (36.4%) patients had received one kind of TKI as previous treatment, while another eight (36.4%) patients, six (27.2%) patients had received two and three or more TKIs, respectively. For anti-PD-1 inhibitors, the number of patients receiving pembrolizumab, sintilimab, camrelizumab, or tislelizumab was three (13.6%), six (27.3%), five (22.7%), and eight (36.4%), respectively. Most patients (21/22, 95.4%) received anti-PD-1 inhibitors in combination with chemotherapy/angiogenesis.

**Table 1:** Baseline characteristics.

Baseline characteristics	All patients (n = 22)
Gender, n (%)	
Male	10 (45.5)
Female	12 (54.5)
Age	
Median (range)	58 (42–83)
Histological subtypes, n (%)	
Adenocarcinoma	20 (90.9)
Squamous cell cancer	2 (9.1)
ECOG status, n (%)	
0	14 (63.6)
1	7 (31.8)
2	1 (4.6)
Smoking status, n (%)	
Never smoked	9 (40.9)
Current or former smoker	13 (59.1)
BMI index, n (%)	
< 18.5	2 (9.1)
18.5–23.9	15 (68.2)
≥ 24.0	5 (22.7)
EGFR mutations, n (%)	
Exon 19 deletion	12 (54.5)
Exon 21 L858R mutation	10 (45.5)
Previous kinds of TKIs, n (%)	
1	8 (36.4)
2	8 (36.4)
≥ 3	6 (27.2)
Acquired T790M mutations, n (%)	
Yes	12 (54.6)
No	5 (22.7)
Unknown	5 (22.7)
Anti-PD-1 inhibitors, n (%)	
Pembrolizumab	3 (13.6)
Sintilimab	6 (27.3)
Camrelizumab	5 (22.7)
Tislelizumab	8 (36.4)
PD-1 combined/alone, n (%)	
Combined	21 (95.4)
Alone	1 (4.6)
Previous radiotherapy, n (%)	
Yes	9 (40.9)
No	13 (59.1)

ECOG, eastern cooperative oncology group; EGFR, epidermal growth factor receptor; TKI, tyrosine kinase inhibitor; PD-1, programmed death 1.

### Clinical outcomes

**Figure 1:**
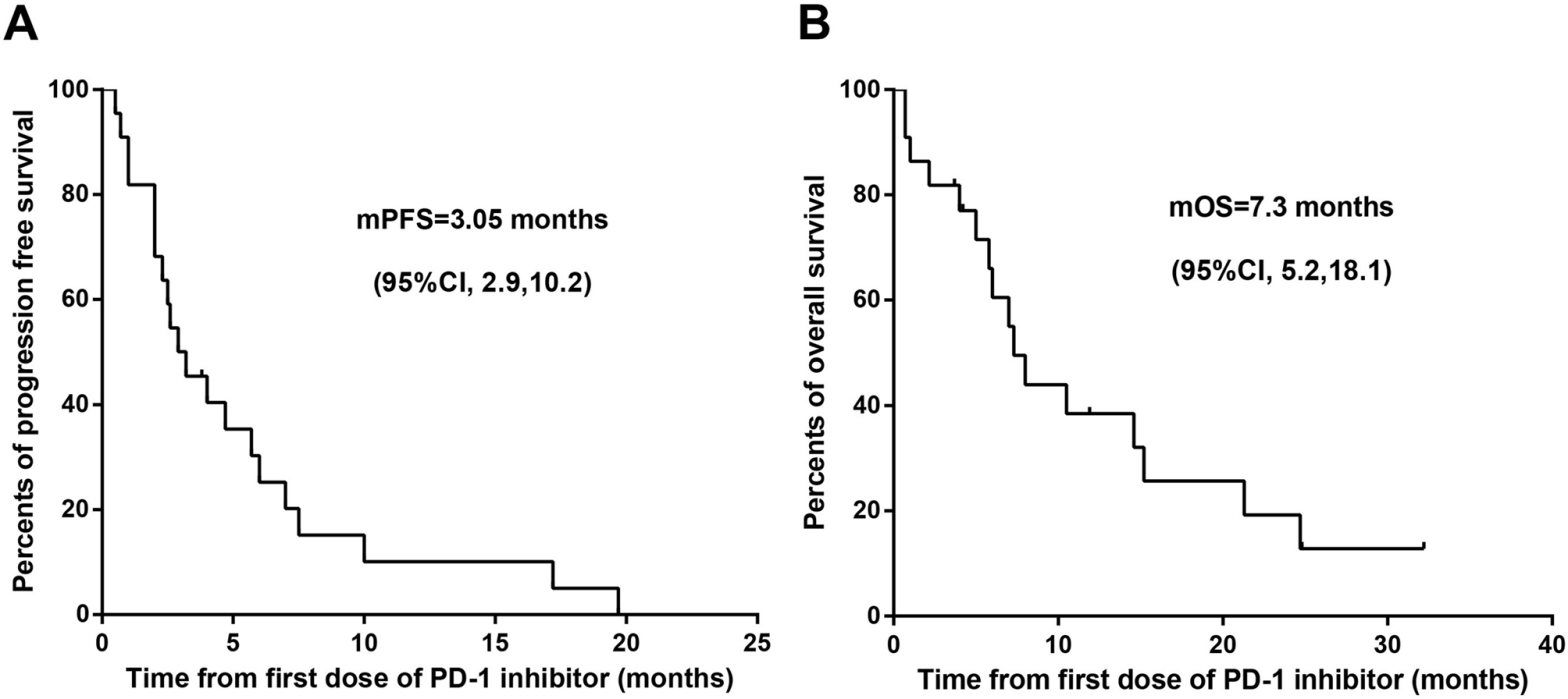
Kaplan–Meier survival curves of PFS (**A**) and OS (**B**) in 22 patients. PFS, progression-free survival; OS, over survival; CI, confidence interval; PD-1, programmed death 1.

During treatment with anti-PD-1 inhibitors, 20 patients underwent at least one imaging evaluation. As presented in Table 2, ORRs were observed in 3 of 20 (15.0%) patients, and the DCR was observed in 15 of 20 (75.0%) patients. The median DoR among the three patients was 7.0 months (95%CI, 6.0, 7.5). The median PFS was 3.05 months with 95% CI of 2.9–10.2 months (Fig. 1A). The median OS was 7.30 months with 95% CI of 5.2–18.1 months (Fig. 1B).

**Table 2:** Efficacy of anti-PD-1 inhibitors in EGFR-TKI-resistant non-small cell lung cancer (n = 20).

Efficacy	All patients (n = 20)
Complete response (%)	0 (0)
Partial response (%)	3 (15.0)
Stable disease (%)	12 (60.0)
Progressive disease (%)	5 (25.0)
Objective response rate (%, CR, PR)	3 (15.0)
Disease control rate (%, CR, PR, SD)	15 (75.0)
mDOR (months, n = 3)	7.0 (6, 7.5)
mPFS (months, 95% CI)	3.05 (2.9, 10.2)
mOS (months, 95% CI)	7.30 (5.2, 18.1)

PD-1, programmed death 1; EGFR, epidermal growth factor receptor; TKI, tyrosine kinase inhibitor; DOR, duration of response; mPFS, median progression-free survival; mOS, median overall survival.

### Subgroup analysis and multivariate analysis

**Figure 2:**
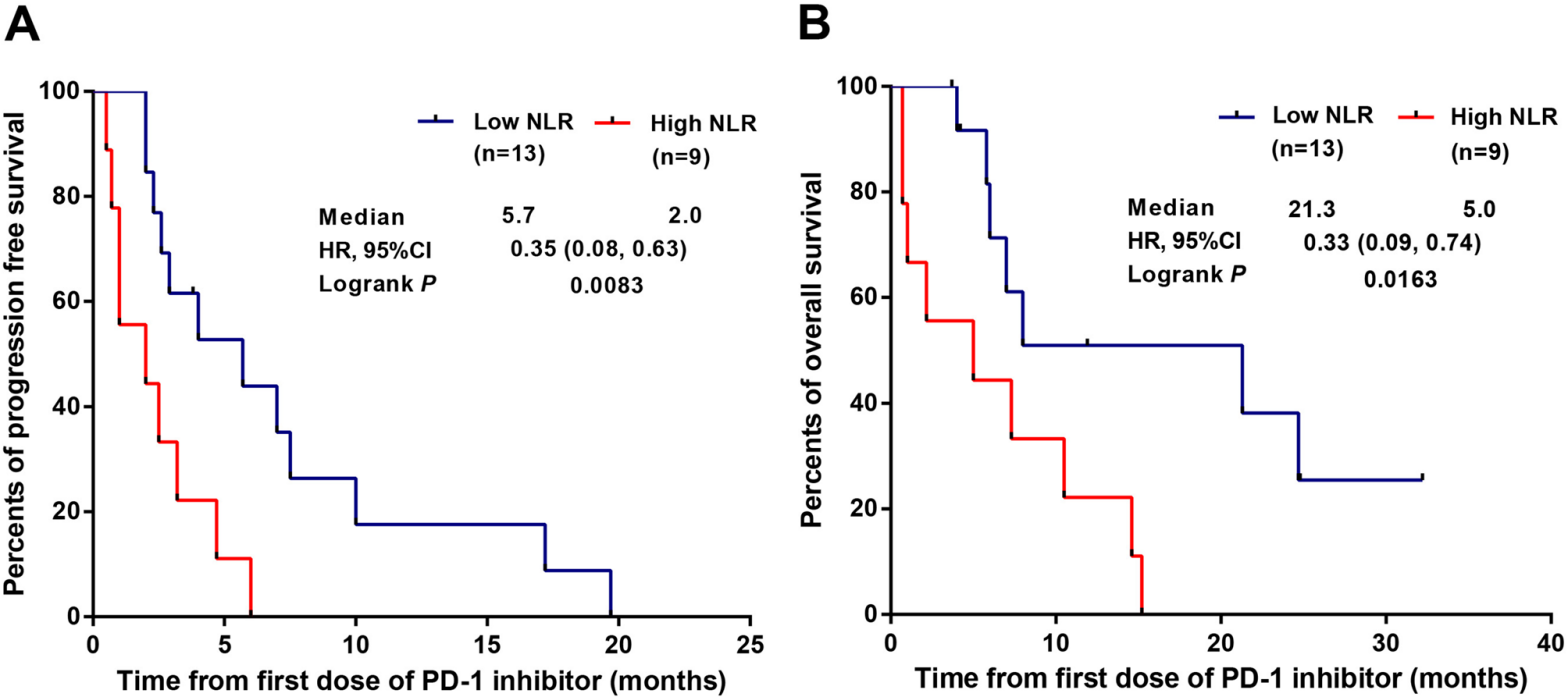
Comparisons for Kaplan–Meier survival curves of progression-free survival (**A**) and overall survival (**B**) between high NLR group (NLR > 4, n = 9) and low NLR group (NLR ≤ 4, n = 13). NLR, neutrophil to lymphocyte ratio; CI, confidence interval; PD-1, programmed death 1.

All patients were categorized into the high NLR group (NLR > 4, n = 9) and low NLR group (NLR ≤ 4, n = 13) according to the parameters tested two weeks before the initiation of the first dose of anti-PD-1 inhibitors. As presented in Fig. 2A, PFS in the low NLR group was significantly longer than that in the high NLR group (PFS, 5.7 months versus 2.0 months, HR, 0.35; 95% CI, 0.08–0.63;*P* = 0.0083). The OS in the low NLR group was also significantly better than that in the high NLR group (OS, 21.3 months versus 5.0 months, HR, 0.33; 95% CI, 0.09–0.74; *P* = 0.0163, Fig. 2B).

In multivariate analysis showed in Table 3, the significant factors for PFS included NLR (β = 5.275, 95% CI, 1.703–16.341, *P* = 0.004), BMI (β = 0.329, 95% CI, 0.150–0.724, *P* = 0.006), and sex (β = 5.750, 95% CI, 1.676–19.729, *P* = 0.005). No significant difference on PFS was observed in smoking status (β = 0.615, 95% CI, 0.114–3.310, *P* = 0.572), EGFR mutation types (β = 1.001, 95% CI, 0.338–2.964, *P* = 0.999), TKI-PFS (β = 1.372, 95% CI, 0.477–3.948, *P* = 0.558), numbers of TKIs (β = 0.673, 95% CI, 0.217–2.514, *P* = 0.429), or with or without T790M mutation (β = 0.831, 95% CI, 0.243–2.845, *P* = 0.768). In the multivariate analysis for OS, NLR was the only significant factor detected for OS analysis (β = 3.535, 95% CI, 1.175–10.636, *P* = 0.025).

**Table 3:** Multiple regression analysis to elucidate clinical variables associated with PFS and OS.

	PFS		95% CI		OS		95% CI	
	β	*P*			β	*P*		
Gender	5.750	0.005	1.676	19.729	1.863	0.250	0.645	5.376
Smoking	0.615	0.572	0.114	3.310	0.474	0.400	0.083	2.701
BMI	0.329	0.006	0.150	0.724	1.026	0.952	0.447	2.356
EGFR mutations	1.001	0.999	0.338	2.964	0.588	0.442	0.152	2.276
T790M	0.831	0.768	0.243	2.845	0.590	0.445	0.152	2.286
Numbers of TKIs	0.673	0.429	0.217	2.514	0.471	0.524	0.214	3.493
NLR	5.275	0.004	1.703	16.341	3.535	0.025	1.175	10.636

Shown are standard regression coefficient values (β values) and level of significance.

PFS, progression free survival; OS, overall survival; BMI, body mass index; EGFR, epidermal growth factor receptor; NLR, neutrophil to lymphocyte ratio.

### Variation of NLR

**Figure 3:**
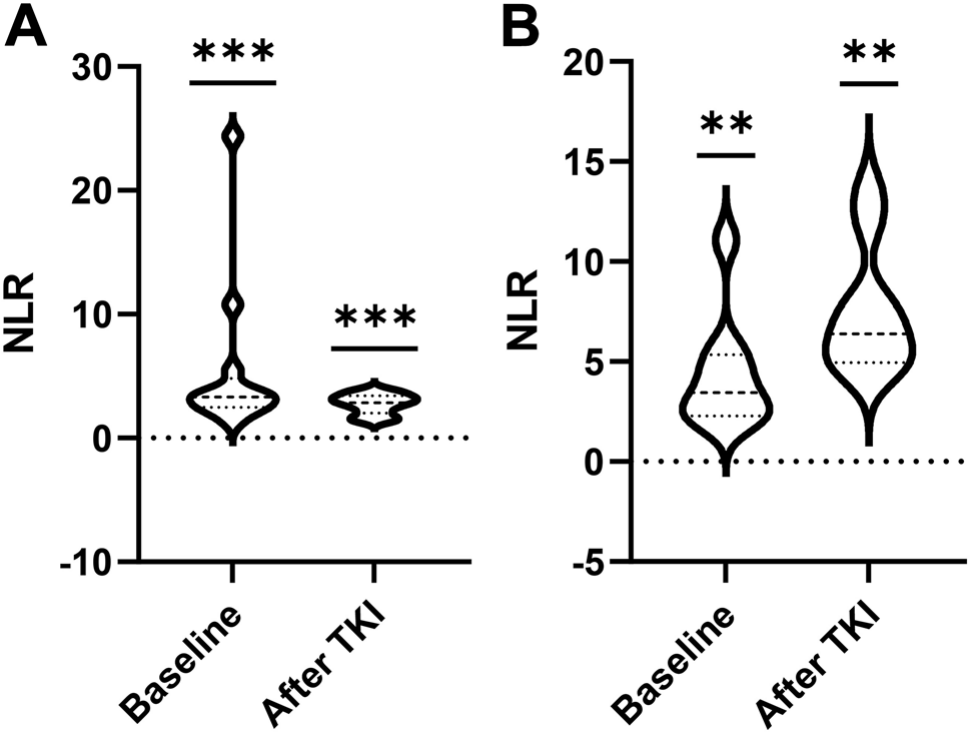
Variation of NLR at the time of the initiation of TKIs (Baseline at the diagnosis of NSCLC) and the time of the beginning of anti-PD-1 inhibitors between the low NLR group (**A**) and high NLR group (**B**). NLR, neutrophil to lymphocyte ratio, TKI, tyrosine kinase inhibitor. **P < 0.001, ***P < 0.0001.

Multivariate analysis showed that NLR during the two weeks before the initiation of anti-PD-1 inhibitors might be an independent prognostic factor for OS benefits of immunotherapy. We further investigated the variation in NLR at the time of the initiation of TKIs (baseline at the diagnosis of NSCLC) and the time of the beginning of anti-PD-1 inhibitors between the low NLR group and the high NLR group, which revealed a significant elevation of NLR in the high NLR group (median NLR, 3.44–6.38, *P* = 0.0039), as well as a significant decrease in NLR in the low NLR group (median NLR, 3.33–2.85, *P* = 0.0002) (Fig. 3).

### Safety

Treatment-related adverse events during the administration of anti-PD-1 inhibitors (in combination with chemotherapy/angiogenesis or alone) are presented in Table 4. The most common grade 1–2 adverse events were anemia 15/22 (68.2%), fatigue 11/22 (50.0%), decreased appetite 9/22 (40.9%), nausea 8/22 (36.4%),andneutropenia7/22 (31.8%). The less common grade 1–2 adverse events included pneumonitis 5/22 (22.7%), abnormal hepatic function 5/22 (22.7%), vomiting 4/22 (18.2%), diarrhea 3/22 (13.6%), thrombocytopenia 3/22 (13.6%), hypothyroidism 3/22 (13.6%), elevated creatinine 2/22 (9.1%), and rash 2/22 (9.1%).

**Table 4:** Adverse events (n = 22).

Adverse events	Grade 1–2, n (%)	Grade 3–4, n (%)
Fatigue	11 (50)	1 (4.5)
Rash	2 (9.1)	0
Decreased appetite	9 (40.9)	1 (4.5)
Nausea	8 (36.4)	0
Vomiting	4 (18.2)	0
Diarrhea	3 (13.6)	0
Anemia	15 (68.2)	0
Neutropenia	7 (31.8)	1 (4.5)
Thrombocytopenia	3 (13.6)	1 (4.5)
Pneumonitis	5 (22.7)	0
Hepatic function abnormal	5 (22.7)	2 (9.1)
Elevated creatinine	2 (9.1)	0
Hypothyroidism	3 (13.6)	0

The grades 3–4 adverse events included hepatic function abnormal in 2/22 (9.1%), neutropenia in 1/22 (4.5%), thrombocytopenia in 1/22 (4.5%), decreased appetite in 1/22 (4.5%), and fatigue in 1/22 (4.5%).

## Discussion

The present study was conducted to investigate the associations between clinical characteristics and the effectiveness of anti-PD-1 inhibitors in patients with advanced NSCLC harboring EGFR-sensitive mutations. Accordingly, the results of this retrospective study demonstrated that patients with low NLR (NLR ≤ 4) before anti-PD-1 inhibitor treatment had significantly prolonged PFS and OS benefits than those with high NLR (NLR > 4). To the best of our knowledge, the present study is the first to report that there may exist a group of patients with low NLR characteristics, even harboring EGFR-sensitive mutations, might benefit from anti-PD-1 inhibitors as further line treatment after progression to TKIs.

Although anti-PD-1 inhibitors have been approved as standard treatment options for advanced NSCLC, their efficacy in patients harboring targetable sensitive mutations such as EGFR or ALK has not been identified. The results of the large real-world retrospective study IMMUNOTARGET showed that the ORRs of ICIs in driver alteration patients were as follows: KRAS, 26%; BRAF, 24%, ROS1 = 17%; MET, 16%; EGFR, 12%, HER2 = 7%; RET, 6%; and ALK, 0%. The median PFS (in months) was 2.1 for EGFR, 3.2 for KRAS, 2.5 for ALK, 3.1 for BRAF, 2.5 for HER2, 2.1 for RET, and 3.4 for MET, suggesting that patients with NSCLC who had actionable tumor alterations poorly responded to immunotherapy, as well as limited efficacy as a single agent^5^. In terms of combinational treatment, the phase III prospective, randomized clinical trial KEYNOTE-789 has released its preliminary results in the most recently, results of which suggested that the addition of pembrolizumab to chemotherapy fail to prolong PFS or OS in TKI-resistant EGFR-mutant NSCLC patients. In addition, there was a retrospective research conducted to identify the characteristics of patients who may respond to anti-PD-1 inhibitors therapy for EGFR-mutant NSCLC, results of which revealed that patients who had responded to EGFR TKIs as front line treatment for more than ten months exhibited a significantly shorter response duration to anti-PD-1 inhibitors compared to those responded for less than ten months (PFS of ICI:1.6 vs. 1.9 months; HR:2.54; 95% CI, 1.26–5.12, *P* = 0.009). Nevertheless, patients who responded to ICIs for more than six months responded to EGFR-TKIs for significantly shorter PFS compared to those less than six months (PFS of EGFR-TKI:5.3 vs. 12.1 months, *P* = 0.0025)^10^. The results of that study suggest that the treatment strategy of TKIs and anti-PD-1 inhibitors may represent opposite and exclusive options. The authors explained that a history of smoking, which potentially increases the tumor mutation burden (TMB), might be associated with a trend of a longer response to anti-PD-1 inhibitors in their cohort^10^. However, this explanation has also been challenged by a different result^11^. In addition, another matched case–control study, which compared the effectiveness of combined an-PD-1 inhibitors with standard chemotherapy as second-line treatment in patients with advanced NSCLC, presented similar results, demonstrating that combined an-PD-1 inhibitors achieved significantly longer PFS (HR, 0.51, 95% CI: 0.31–0.85, *P* = 0.02), longer OS (HR, 0.48, 95% CI: 0.26–0.89, *P* = 0.05), as well as a higher ORR (33.3% vs. 10.0%, *P* = 0.02) than cytotoxic agents for patients with NSCLC who had short TKI-PFS^9^. Furthermore, the authors considered that patients with short TKI-PFS had a higher intratumoral cytotoxic lymphocyte infiltration, as well as a lower rate of M2-like macrophages to M1-like macrophages, which finally contributed to the difference^9^. However, in the present study, we also investigated the association between PFS and efficacy, and the results of the multivariate analysis failed to show a statistical difference in OS (β = 1.372, 95% CI, 0.477–3.948, *P* = 0.558). Although the potential mechanism remains unknown, we considered that the relationship between TKI-PFS and the efficacy of anti-PD-1 inhibitors should be approached prudently. Besides, there were some other studies reported potential correlation between the PD-L1 expression and the efficacy of ICIs^12–14^. However, in the present study, PD-L1 expression was not tested, which led to the difference between NLR and PD-L1 expression. Based on this, we sponsored another prospective real-world study to investigate the predictive value of low NLR in EGFR mutant patients, which included the factor of PD-L1 expression analysis. We hope that these results will address this question in the future.

NLR, a systematic inflammation marker, has been frequently reported as an indicator of systematic response to cancer treatment strategies, including NSCLC^15^. A low NLR was reported to be a positive predictive factor not only for chemotherapy^16^ and radiotherapy^17^, but also for immunotherapy^18–20^. Although the potential mechanism for the correlation between NLR and prognosis is not completely understood, the dominant explanation might be the tumor microenvironment (TME)^20^. Neutrophils are the most abundant immune cell population, traditionally regarded as indispensable antagonists of microbial infection and facilitators of wound healing, and may play a significant role in cancer progression. However, cytotoxic T lymphocyte are critical for mediating cellular immunity against cancer cells. Hence, the ratio of circulating NLR was supposed to correlate with the interaction between systemic inflammation and immunity, especially the potential mechanism in the TME. In the multivariate analysis in the present study, we found that NLR (two weeks before the initiation of anti-PD-1 inhibitors) was the only statistically significant factor for OS (β = 3.535, 95% CI, 1.175–10.636, *P* = 0.025). We speculated that front-line EGFR-TKI treatment might play a role in the alteration of NLR, which contributes to the different outcomes of anti-PD-1 inhibitors. In the variation analysis of NLR, we found that TKIs treatment may lead to an increase in NLR or decrease in NLR, which finally resulted in the opposite efficacy outcomes between the groups (Fig. 3). In our previous study, we also found that NLR could be influenced by antitumor strategies and varied consistently during treatment^21^. It was not a unique instance, another retrospective study, aiming to investigate whether astragalus polysaccharide injection (PG2) might influence the NLR and affect the OS in patients with lung cancer treated with ICIs, presented a result that NLR significantly decreased by 31.60% in the PG2 group (*P* = 0.012), whereas increased by 5.80% in the control (*P* = 0.572). The results of the study suggested that PG2 could normalize the NLR in patients with lung cancer receiving anti-PD-1 combination treatments^20^. A similar clinical phenomenon was observed in patients with cervical cancer undergoing definitive chemoradiotherapy^22^. Hence, the potential hypothesis might be that there exists a percentage of patients with advanced NSCLC, of which the NLR might be decreased by EGFR-TKIs, who may benefit from salvage treatment with anti-PD-1 inhibitors after progression to TKIs. However, because of the limited sample size in the present study, further prospective research with a larger sample size might be essential to identify this clinical phenomenon.

This study had some limitations. Firstly, the retrospective nature of the study with a limited sample size may limited the value of the conclusion. Thus, information bias could not be excluded. Furthermore, PD-L1 expression was not evaluated in the present study. As a significant element may influence the final results, the absence of testing may reduce the reliability of the conclusion in the present study. Moreover, we considered that different EGFR-TKI usage may also affect the NLR value before the initiation of the study. However, we could not investigate the problem in the present study because of the retrospective design and limited sample size. We hope further researches would address the interesting issue. Finally, the study was designed and performed in a single center, which may have resulted in potential objective bias for data collection and final analysis.

## Conclusion

In conclusion, the findings of the present study suggest that a portion of patients with advanced NSCLC with low NLR characteristics (NLR ≤ 4), even those harboring EGFR-sensitive mutations, could benefit from anti-PD-1 inhibitors as further line treatment after progression to EGFR-TKIs.

## Data Availability

All data generated or analyzed during this study are included in this article.
